# Glutathione metabolism-related gene signature predicts prognosis and treatment response in low-grade glioma

**DOI:** 10.18632/aging.205881

**Published:** 2024-05-30

**Authors:** Zaidong Deng, Jing Luo, Jing Ma, Youngnam N. Jin, Yanxun V. Yu

**Affiliations:** 1 Department of Neurology, Medical Research Institute, Zhongnan Hospital of Wuhan University, Wuhan University, Wuhan, China; 2 Frontier Science Center for Immunology and Metabolism, Wuhan University, Wuhan, China

**Keywords:** low-grade glioma (LGG), glutathione metabolism, risk signature, prognosis, cancer treatment

## Abstract

Cancer cells can induce molecular changes that reshape cellular metabolism, creating specific vulnerabilities for targeted therapeutic interventions. Given the importance of reactive oxygen species (ROS) in tumor development and drug resistance, and the abundance of reduced glutathione (GSH) as the primary cellular antioxidant, we examined an integrated panel of 56 glutathione metabolism-related genes (GMRGs) across diverse cancer types. This analysis revealed a remarkable association between GMRGs and low-grade glioma (LGG) survival. Unsupervised clustering and a GMRGs-based risk score (GS) categorized LGG patients into two groups, linking elevated glutathione metabolism to poorer prognosis and treatment outcomes. Our GS model outperformed established clinical prognostic factors, acting as an independent prognostic factor. GS also exhibited correlations with pro-tumor M2 macrophage infiltration, upregulated immunosuppressive genes, and diminished responses to various cancer therapies. Experimental validation in glioma cell lines confirmed the critical role of glutathione metabolism in glioma cell proliferation and chemoresistance. Our findings highlight the presence of a unique metabolic susceptibility in LGG and introduce a novel GS system as a highly effective tool for predicting the prognosis of LGG.

## INTRODUCTION

Glioma is one of the most prevalent malignant tumors affecting the central nervous system, characterized by aggressive growth, high resistance to therapy, and increased recurrence rates [1–4]. The 2021 World Health Organization (WHO) classification criteria categorize malignant gliomas into low-grade gliomas (WHO grade II and III) and high-grade gliomas, notably glioblastoma (GBM, WHO IV) [5]. Low-grade gliomas (LGG) exhibit clinical and histological heterogeneity, with varying prognoses and risks of progression to aggressive GBM [6, 7]. Despite traditional approaches such as surgery, radiotherapy, and chemotherapy, treatment options for LGGs are limited, resulting in frequent disability and premature death.

The current treatment strategy for LGG involves a comprehensive assessment of various prognostic factors, encompassing age, gender, initial symptoms, neurological deficits, Karnofsky Performance Status (KPS), tumor grade, histology, tumor size and localization, and the extent of resection [8–12]. While molecular markers such as IDH mutations, 1p19q co-deletions, and MGMT promoter methylation play a crucial role in prognosis, diagnosis, management, and potential targeted treatments of LGG [13–18], their limitations are evident, particularly given the prevalence of IDH1 mutations in LGG patients [19–21].

Reactive oxygen species (ROS) play an important role in cancer progression [22, 23], with cancer cells frequently displaying elevated baseline ROS due to metabolic shifts and genomic mutations [24, 25]. This requires a higher antioxidant demand for cancer survival [26, 27]. Furthermore, the cancer redox environment constitutes a crucial element of the tumor immune microenvironment (TIME), influencing tumor progression, immune infiltration, immune evasion, and responses to immunotherapy [28, 29]. Reduced glutathione (GSH), as the most abundant intracellular antioxidant, plays a vital role in cellular defense against oxidative stress, contributing to metastasis and chemoresistance [30]. Classical tumor metabolic pathways, including Myc, p53, and PI3K can perturb intracellular glutathione levels [31–33]. The depletion of glutathione has been used in cancer therapy [31, 34, 35]. Despite the crucial role of glutathione metabolism in cancer therapy, the characterization of glutathione metabolism-related signatures in low-grade glioma (LGG) prognosis and treatment remains underexplored.

While previous studies have explored the use of antioxidants, including GSH levels and antioxidant enzymes, to predict LGG prognosis and guide treatment [36–38], a systematic investigation into the collective impact of an integrated GMRGs gene set on LGG prognosis and treatment has been lacking. In our study, we demonstrate that a risk score derived from a set of GMRGs serves as a highly effective predictive factor for LGG prognosis and treatment outcomes. Additionally, we provide experimental evidence from glioma cell lines indicating the critical role of GSH metabolism levels in cancer cell progression.

## RESULTS

**Figure 1:**
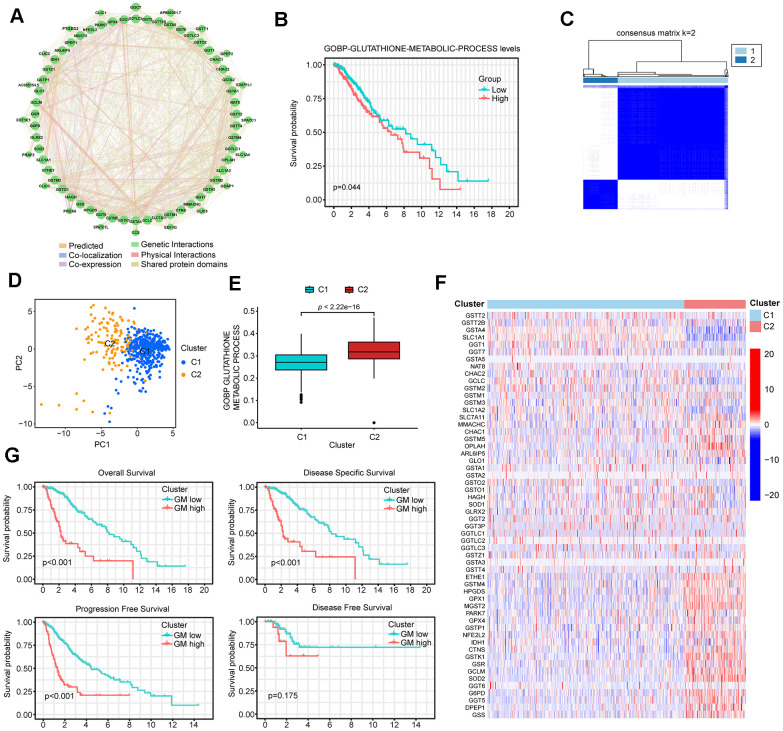
**Identification of two distinct clusters in low-grade glioma (LGG) patients using consensus clustering.** (**A**) The protein-protein interaction (PPI) network of GMRGs. (**B**) Kaplan-Meier (K-M) survival analysis based on glutathione metabolism scores. (**C**) Consensus clustering divides all The Cancer Genome Atlas (TCGA)-LGG patient samples into two clusters. (**D**) PCA analysis shows that the two GMRGs-related clusters are distinctly separated. (**E**) Glutathione metabolism differs between the two clusters. (**F**) Glutathione metabolism related genes (GMRGs) expression levels in the two clusters. (**G**) Survival differences between the two clusters in OS, disease-specific survival (DSS), progression-free survival (PFS), and disease-free survival (DFS).

Given the important role of glutathione metabolism in tumor development, our study commenced by identifying all 56 human GMRGs from the MSigDB database using the gene set “GOBP_GLUTATHIONE_METABOLIC_PROCESS”. Subsequently, we examined the expression levels of these GMRGs and their correlation with patient prognosis across 33 different cancer types, encompassing more than 10,000 patient samples. When ranked by survival, LGG exhibited a strong association of patient survival with this gene set, with almost half of the genes significantly associated with overall survival (OS) in LGG patients. (Supplementary Figure 1 and Supplementary Table 1). Among the 56 GMRG genes, 35 are up-regulated and 17 are down-regulated in LGG patient samples compared to normal brain tissue (Supplementary Figure 2A). Additionally, GMRGs were found to be associated with infrequent mutations (Supplementary Figure 2B), but extensive copy number alterations (Supplementary Figure 2C). A genetic interaction network was constructed with GeneMANIA which demonstrated the association among GMRGs (Figure 1A).

**Figure 2:**
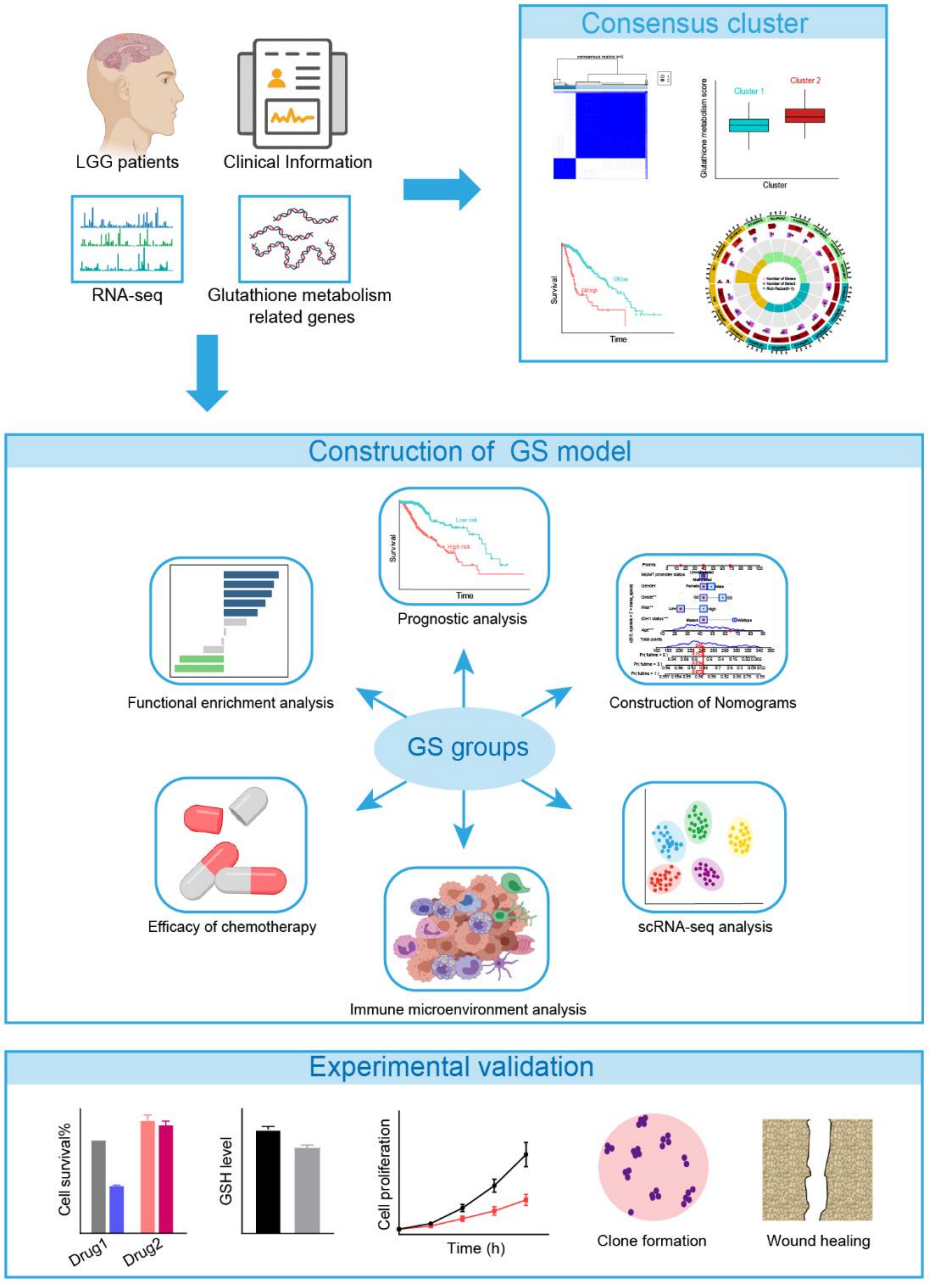
The flowchart of the study.

Next, we assessed the impact of glutathione metabolism on survival in LGG patients and discovered a significant correlation between high glutathione metabolism and worse survival rates (Figure 1B). These findings suggest that glutathione metabolic processes play an important role in the development and prognosis of LGG. To effectively utilize the predictive power of GMRGs in LGG treatment and prognosis, we implemented two modeling approaches. Additionally, we validated treatment paradigms and key gene functions in glioma cell lines. The workflow of the subsequent study is depicted in Figure 2.

### Consensus clustering analysis of GMRGs

### Identification of two GMRGs-related clusters

To identify distinct subgroups of LGG patients based on their GMRGs expression patterns, we employed consensus clustering. This analysis revealed 2 distinct clusters, designated as Cluster 1 (n=392) and Clusters 2 (n=122) (Figure 1C). Principal Component Analysis (PCA) further confirmed the distinct separation of LGG patients into these two clusters (Figure 1D). Cluster 2 exhibited significantly higher glutathione metabolism scores compared to Cluster 1 (Figure 1E). The heatmap further illustrated that Cluster 2 has higher GMRGs expression levels compared to Cluster 1 (Figure 1F). Therefore, we designated Cluster 2 as the glutathione metabolism-high cluster (GM-high cluster) and Cluster 1 as the glutathione metabolism-low cluster (GM-low cluster). Survival analysis revealed that GM-low cluster patients had a significantly superior prognosis for overall survival (OS), disease-specific survival (DSS), progression-free survival (PFS). While disease-free survival (DFS) displays a tendency to favor GM-low cluster patients, the difference is not statistically significant (Figure 1G). These findings highlight the strong association between GMRGs-related clusters, glutathione metabolic activity, and prognosis in LGG patients.

### Functional analysis of the two GMRGs-related clusters

To explore the molecular and cellular mechanisms underlying the disparate clinical outcomes observed in the two clusters, we performed functional enrichment analysis. Employing stringent criteria (| logFoldChange| >1 and FDR < 0.05), we identified 3,898 Differentially Expressed Genes (DEGs) between the two clusters (Supplementary Table 2). Gene Ontology (GO) functional enrichment analysis revealed significant enrichment of these DEGs in leukocyte-mediated immunity, antigen binding, external side of plasma membrane, and MHC protein complex (Supplementary Figure 3A). Kyoto Encyclopedia of Genes and Genomes (KEGG) enrichment analysis further highlighted the association of DEGs with neuroactive ligand−receptor interaction, cytokine−cytokine receptor interaction, focal adhesion, and the natural killer cell-mediated cytotoxicity (Supplementary Figure 3B). These findings suggest that the immune microenvironment may play a crucial role in shaping the distinct phenotypes of the clusters.

### Development and validation of a GMRGs-based risk score model

### Development of GMRGs-based risk score (GS)

**Figure 3:**
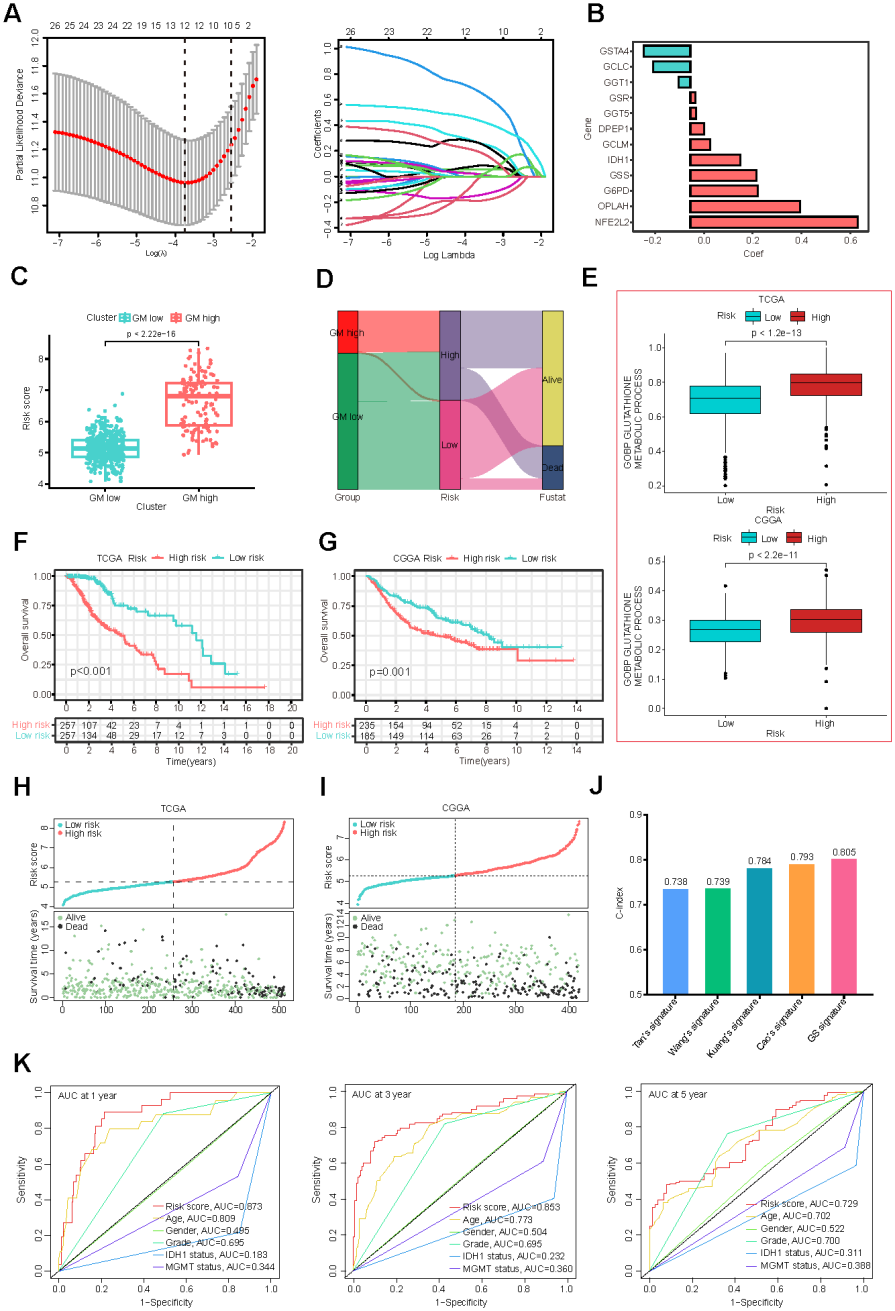
**Construction and validation of the GMRGs-based risk score.** (**A**) Lasso Cox regression analysis identified 12 prognostic GMRGs for signature construction. (**B**) Coefficients for the 12 genes. (**C**) Risk scores correlate with the two GMRGs-related clusters from consensus clustering. (**D**) The Sankey diagram demonstrates the association of GMRGs-related clusters with GMRGs-based risk score and the survival status of LGG patients. (**E**) Glutathione metabolism scores are associated with high-risk and low-risk groups in the TCGA-LGG and CGGA-LGG datasets. (**F**, **G**) OS curves between the high-risk and low-risk groups in the TCGA-LGG and CGGA-LGG datasets. (**H**, **I**) Distribution map of risk scores (top) and patient survival status (bottom) in the TCGA-LGG and CGGA-LGG datasets. (**J**) The C-index of, Tan’s, Wang’s, Kuang’s, Cao’s and our GMRGs-based risk score (GS) signatures. (**K**) ROC curves comparing risk score, age, gender, grade, IDH1 status, and MGMT promoter status.

To develop a more robust GMRGs-based prognostic model, three machine learning methods (Lasso, SuperPC, plsRcox) were used for model construction (Figure 3A and Supplementary Figure 4A, 4B). The intersection of the model genes screened by the three methods revealed that the 12 genes identified by the Lasso method were also identified by the other two methods (Supplementary Figure 4C and Supplementary Table 3). We compared the C-index of the models constructed by three methods and found that the Lasso method exhibited the highest C-index (Supplementary Figure 4D). Therefore, we employed LASSO Cox regression analysis to construct our model and identified 12 genes for inclusion in the predictive risk model based on the optimized λ score (Figure 3A). Employing gene expression levels and regression coefficients, we formulated the risk score as follows: risk score = (0.0560)*DPEP1 + (0.2753)*G6PD + (-0.1521)*GCLC + (0.0802)*GCLM + (-0.0492)*GGT1 + (0.0231)*GGT5 + (0.0213)*GSR + (0.2717)*GSS + (-0.1901)*GSTA4 + (0.2046)*IDH1 + (0.6843)*NFE2L2 + (0.4495)*OPLAH (Figure 3B). The expression levels of these 12 genes among TCGA-LGG tumor samples and CCLE CNS/Brain cell lines were illustrated (Supplementary Figure 4E, 4F). We also explored the perturbation effects of these genes which suggested that the importance of DPEP1, G6PD, GGT1, GGT5, IDH1, and NFE2L2 for glioma cell survival (Supplementary Figure 4G). This formula revealed higher risk scores for the GM-high cluster (Figure 3C), indicating a strong correlation between the consensus clustering and the risk score. The Sankey diagram (Figure 3D) further corroborates this association. Additionally, the high-risk group exhibited elevated glutathione metabolism scores (Figure 3E), similar to the GM-high cluster (Figure 1E).

### Validation of the GMRGs-based risk model

Survival analysis based on the GS model revealed that both TCGA and CGGA patients in the low-risk group experienced a more favorable prognosis compared to those in the high-risk group (Figure 3F, 3G). A positive correlation was also observed between risk scores and mortality rates, as evidenced by the distribution of risk scores and clinical outcomes (Figure 3H, 3I). Additionally, for patients who received radiotherapy or chemotherapy in both datasets, higher risk scores were associated with poorer outcomes (Supplementary Figure 4H, 4I), suggesting that the GS model is also useful in predicting the prognosis of radiotherapy and chemotherapy in LGG patients.

When assessed using a univariate Cox analysis, the GS model demonstrated the strongest association between GS and LGG prognosis among 33 different cancer types (Supplementary Figure 4J). Additionally, the GS score is significantly associated with prognosis in other cancer types, including liver hepatocellular carcinoma (LIHC), acute myeloid leukemia (LAML), prostate adenocarcinoma (PAAD), uveal melanoma (UVM), glioblastoma multiforme (GBM), and uterine carcinosarcoma (UCS) (Supplementary Figure 4J), suggesting that it may be a useful prognosis tool for a variety of cancers. Compared to existing LGG prognostic models [39–42], the GS model exhibited superior predictive performance, as evidenced by higher-index values (Figure 3J). The GS model also outperformed established clinical prognostic factors, including neoplasm grade, IDH1 mutation status, and MGMT promoter methylation, as indicated by higher area-under-the-curve (AUC) values in ROC curves (Figure 3K).

### Independent prognostic analysis and nomogram construction

**Figure 4:**
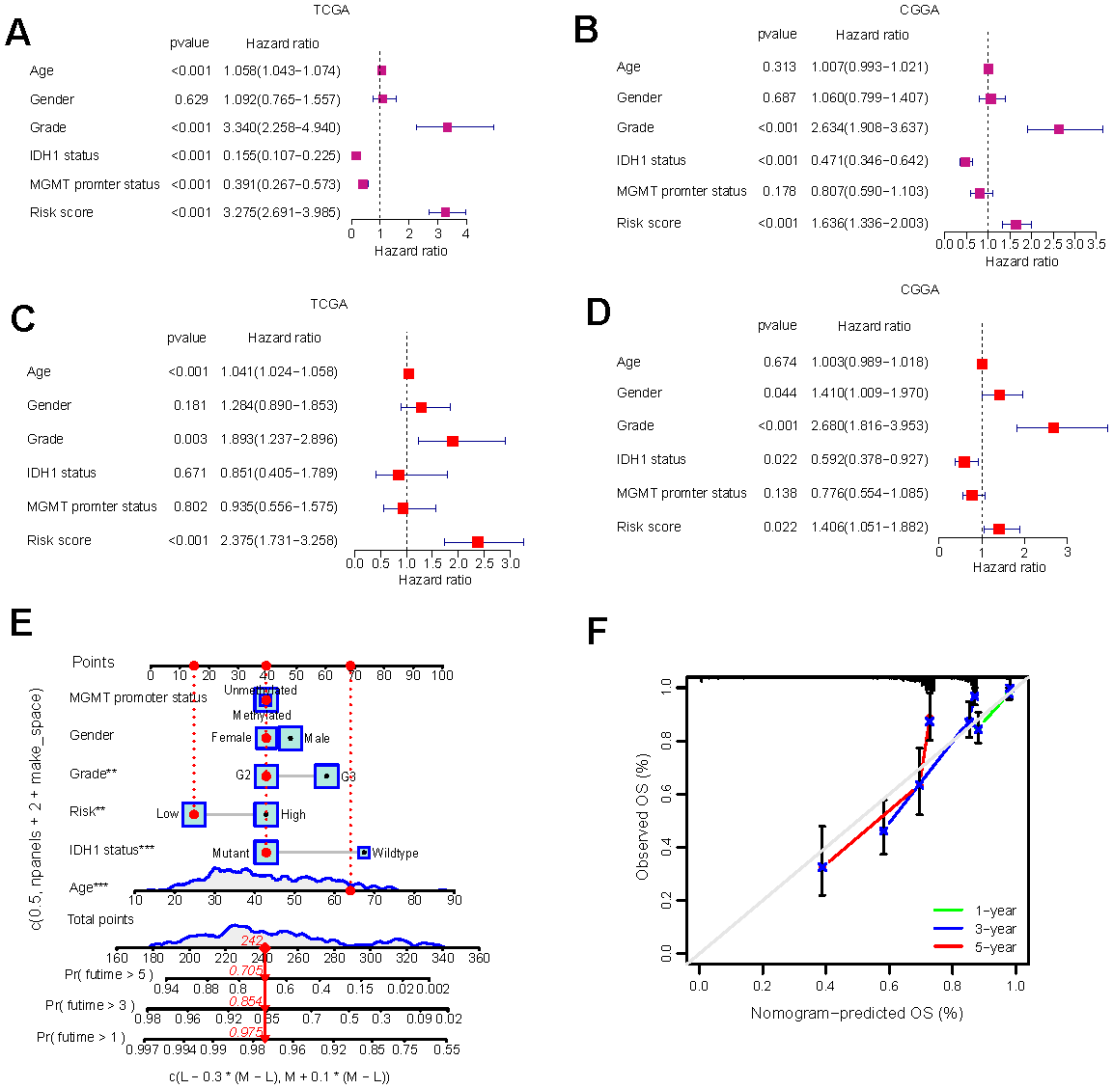
**Independent prognostic analysis and the construction of nomogram.** (**A**–**D**) Univariate Cox regression (**A**, **B**) and multivariate Cox regression (**C**, **D**) analysis of risk scores based on OS in TCGA-LGG and CGGA-LGG datasets. (**E**) Nomograms incorporating the GS and several clinical characteristics to predict the survival probability of LGG patients. (**F**) Calibration curves of the nomogram at 1-, 3-, and 5-year intervals.

Following the construction of the predictive risk model, we investigated whether our GS scoring system overlaps with other known clinical factors. Univariate Cox analyses indicated that the GS risk score, neoplasm grade, IDH1 mutation status, and MGMT promoter methylation were all significantly prognostic factors (Figure 4A, 4B). Significant relationships were also observed between the GS risk score and each of these clinical factors (Supplementary Figure 5A–5D). However, multivariate Cox analyses demonstrated that the GS risk score remained an independent prognostic factor for LGG prognosis even after adjusting for other clinical factors (Figure 4C, 4D).

To enable quantitative predictions in LGG patients, we developed an innovative nomogram based on risk scores and clinical variables (Figure 4E). The predicted 1, 3, and 5-year survival rates closely mirrored the actual observations (Figure 4F). The development of the nomogram further validated the risk model and underscored its potential as a valuable tool for risk stratification and clinical decision-making in LGG management.

### Distinct biological behaviors in high-risk and low-risk groups

### Assessment of GS in biological processes and cancer stemness

**Figure 5:**
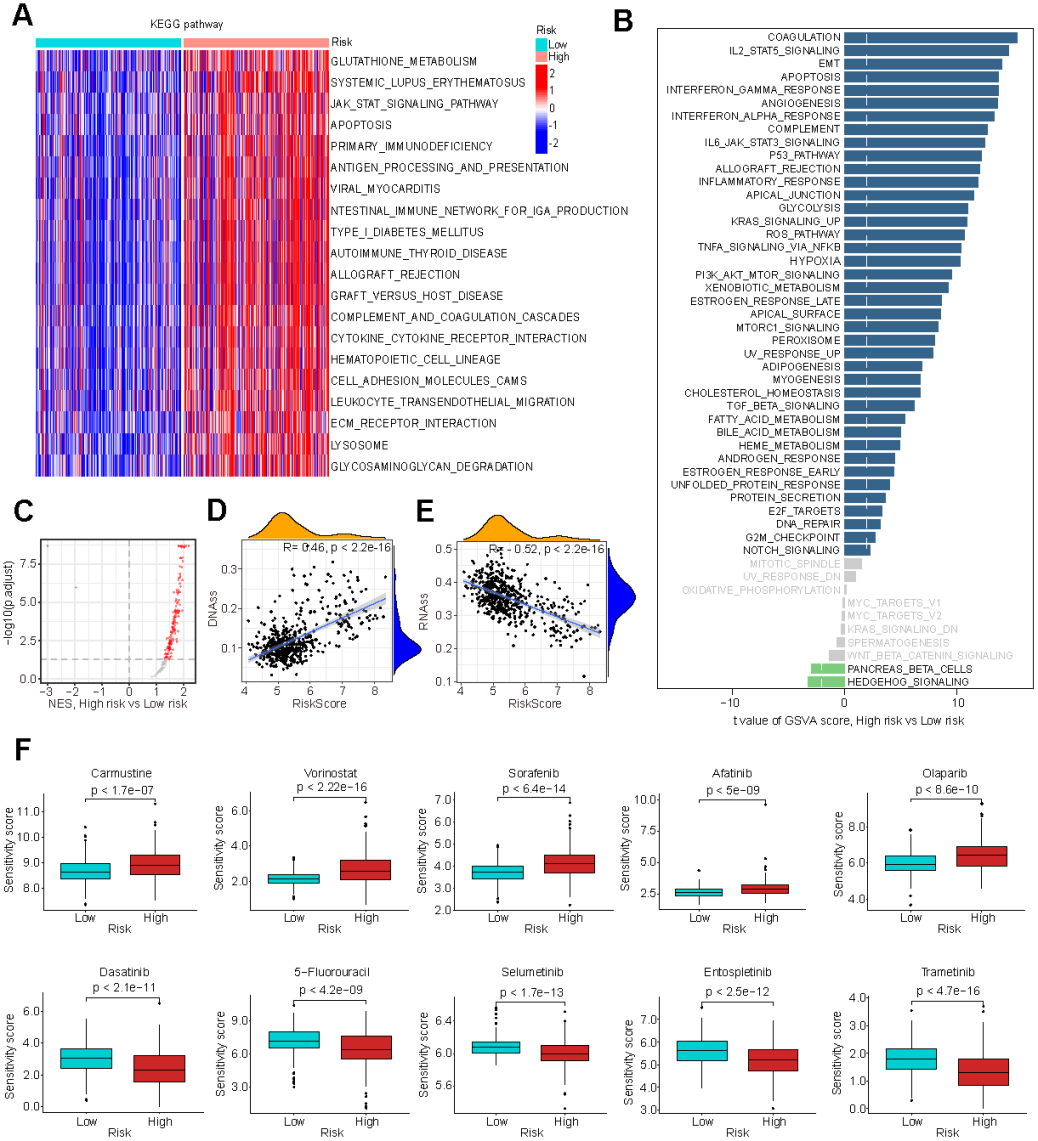
**Biological processes associated with GMRGs-based risk score.** (**A**) Gene set variation analysis (GSVA) depicts KEGG pathway activity differences between high-risk and low-risk groups. (**B**) GSVA reveals activity variation in 50 signature pathways between high-risk and low-risk groups. (**C**) Gene Set Enrichment Analysis (GSEA) illustrates normalized enrichment score (NES) for oncogenic signature gene sets between the high-risk and low-risk groups. (**D**, **E**) Correlation analysis of GS and tumor stem cell index based on DNAss (**D**) and RNAss (**E**). (**F**) Estimation of chemotherapy response for 10 potential therapeutic drugs between high-risk and low-risk groups.

To elucidate the underlying mechanisms by which glutathione metabolism influences LGG cancer prognosis, we investigated its association with biological processes and cancer stemness. KEGG pathway analysis revealed that the high-risk group exhibited upregulation of glutathione metabolism and a variety of immunomodulation-related pathways, such as antigen processing and presentation, cytokine-cytokine receptor interaction (Figure 5A). HALLMARK gene set analysis demonstrated heightened activity in pathways related to ROS, immune responses, and several cancer-promoting pathways, including angiogenesis and epithelial-mesenchymal transition (EMT) (Figure 5B). GSEA revealed that a significant number of oncogenic signature pathways exhibited higher Normalized Enrichment Scores (NES) in the high-risk group (Figure 5C and Supplementary Table 4). Furthermore, we examined the GS**’**s relationship with cancer stem cell traits, uncovering a significant positive correlation with DNA methylation-based stemness score (DNAss) and a significant negative correlation with RNA methylation-based stemness score (RNAss), consistent with previous findings [42, 43] (Figure 5D, 5E).

### Evaluation of GS in chemotherapy efficacy

To further investigate the clinical implications of GS in precision chemotherapy, we evaluated the therapeutic efficacy of various conventional chemotherapy drugs for treating LGG across the two different risk groups. Higher drug sensitivity scores indicate greater resistance to drugs [44]. The findings revealed that individuals in the low-risk group exhibited significantly enhanced responsiveness to Carmustine, Vorinostat, Sorafenib, Afatinib and Olaparib, while demonstrating significantly reduced responsiveness to Dasatinib, 5-Fluorouracil, Selumetinib, Entospletinib and Trametinib with high significance (Figure 5F). These results suggest that GS-based stratification can guide chemotherapy treatment selection to optimize treatment outcomes.

### Exploration of GS and tumor immune microenvironment

**Figure 6:**
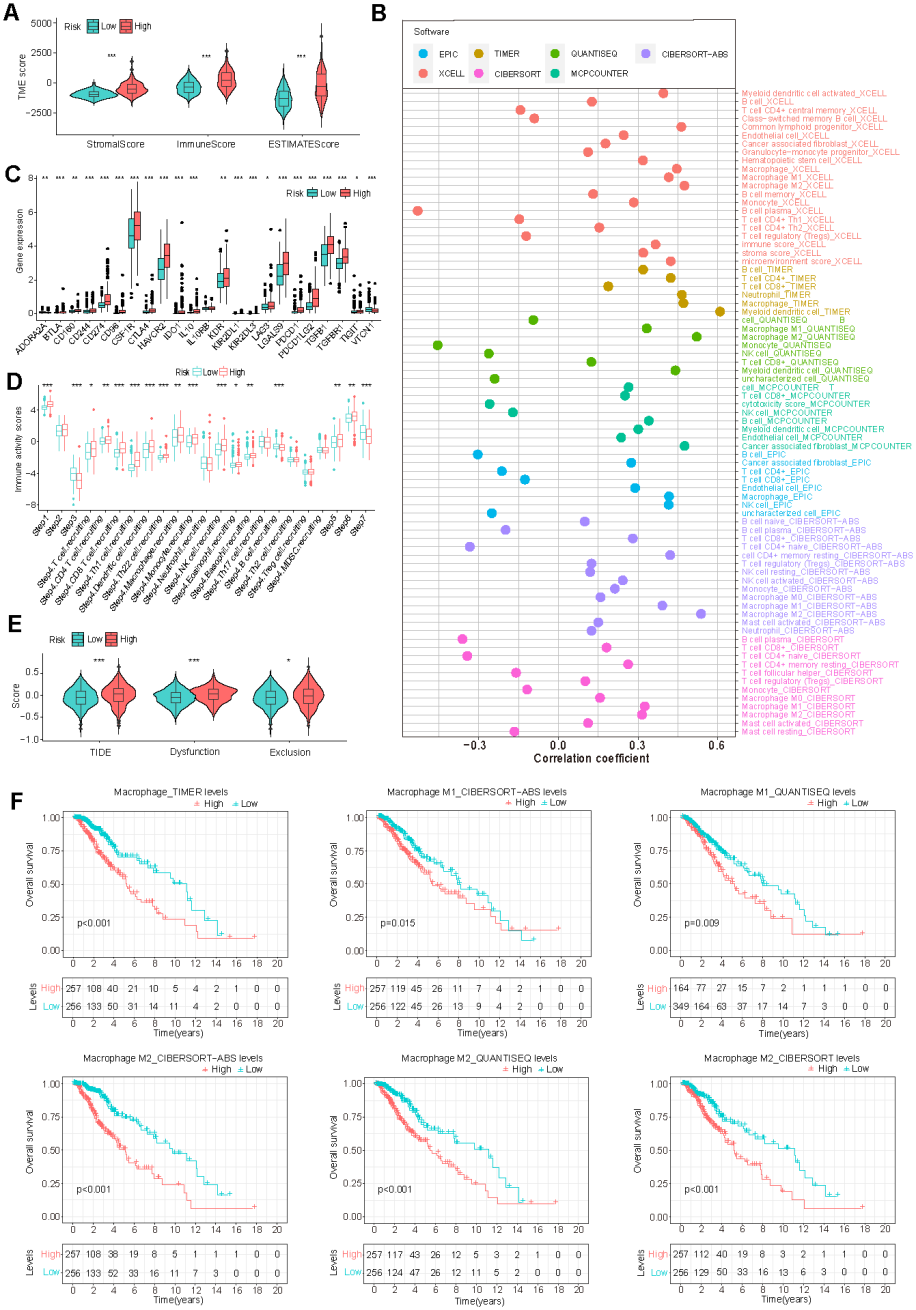
**Correlation of GS and tumor immune microenvironment (TIME) characteristics.** (**A**) Variation in TIME scores between high-risk and low-risk groups. (**B**) Correlation between GS and immune cell infiltration assessed through different immunocyte analysis. (**C**, **D**) Differences in the expression of immunosuppressive genes (**C**) and cancer immune cycle scores (**D**) between high-risk and low-risk groups. (**E**) Disparity in Tumor Immune Dysfunction and Exclusion (TIDE) scores between low-risk and high-risk groups. (**F**) K-M survival analysis based on macrophage infiltration levels in TCGA-LGG patients.

Cancer prognosis is significantly influenced by its immune microenvironment. Therefore, we conducted a comprehensive investigation of immune-related disparities within the TCGA-LGG dataset. Our analysis revealed significant correlations between risk scores and stromal scores, immune scores, and ESTIMATE scores (Figure 6A). Multiple immune cell infiltration analysis methods demonstrated a positive correlation between the risk score and the presence of immune cells such as myeloid dendritic cells, M2 macrophages, and cancer-associated fibroblasts (Figure 6B), which all contribute to tumor prognosis [45–48].

Furthermore, we observed a significant elevation of numerous immune inhibitors in the high-risk group (Figure 6C). This group exhibited increased activity in steps related to antigen release (step 1), T cell transfer (step 4), immune cell infiltration (step 5), and T cell recognition (step 6), while simultaneously demonstrating decreased activity in priming and activation (step 3) and cancer cell killing (step 7) (Figure 6D). These findings provide insights into the intricate immune landscape associated with GS and may help to explain the observed survival differences between risk groups.

Given the apparent connection between GS and the immune microenvironment, we investigated the association between GS and immunotherapy response. The results indicated that the high-risk group had higher TIDE, Dysfunction, and Exclusion scores, suggesting an increased likelihood of immune escape (Figure 6E). Consistent with these findings, our analysis also revealed a correlation between high levels of immune-suppressive macrophage infiltration and poor prognosis in LGG patients (Figure 6F). Additionally, patients in the high-risk group exhibited a worse prognosis in the IMvigor210 immunotherapy cohort (Supplementary Figure 5E). These findings suggest that patients in the high-risk group may be less likely to benefit from immunotherapy if it becomes available for LGG treatment.

### Analysis of GMRGs expression patterns with scRNA-seq

Finally, we conducted an analysis using three glioma single-cell datasets, Glioma_GSE89567, Glioma GSE70630 and Glioma_GSE131928_10X, to examine the cell-specific expression of GMRGs. In the first two datasets, the UMAP plot revealed the presence of four distinct cell populations: astrocytic differentiation (AC)-like malignant cells, mono/macro cells, oligodendrocyte differentiation (OC)-like malignant cells and oligodendrocytes (Supplementary Figure 6A, 7A). We subsequently investigated the expression of model genes in these two datasets. GSTA4 and IDH1 were predominantly expressed in malignant cells, while GSR, GSS, NFE2L2, G6PD, GCLC, GCLM, and GGT1 were expressed in both normal and malignant cells (Supplementary Figures 6B–6D, 7B–7D). In the GSE131928 dataset, eight cell types were clustered, including AC-like Malignant, CD8Tex, MES-like Malignant, Malignant, Mono/Macro, NPC-like Malignant, OPC-like Malignant, and Oligodendrocyte. The expression patterns of the model genes were similar to the GSE89567 and GSE70630 datasets. GSTA4 and IDH1 were predominantly expressed in malignant cells, while GSR, GSS, NFE2L2, G6PD, GCLC, GCLM, and GGT1 were expressed in both normal and malignant cells (Supplementary Figure 8). These findings suggest that some GMRGs are expressed in tumor surrounding cells to play their regulatory roles in cancer progression.

### GSH depletion inhibits glioma cell proliferation and enhances chemotherapy sensitivity

We have demonstrated through comprehensive bioinformatics analyses that GMRGs-related clusters and GS are strongly correlated with glutathione metabolic activity and, in turn, associated with disease treatment and prognosis. To further validate the role of GSH metabolism, we conducted experiments to investigate whether glutathione is essential for proliferation and chemosensitivity in glioma cell lines.

**Figure 7:**
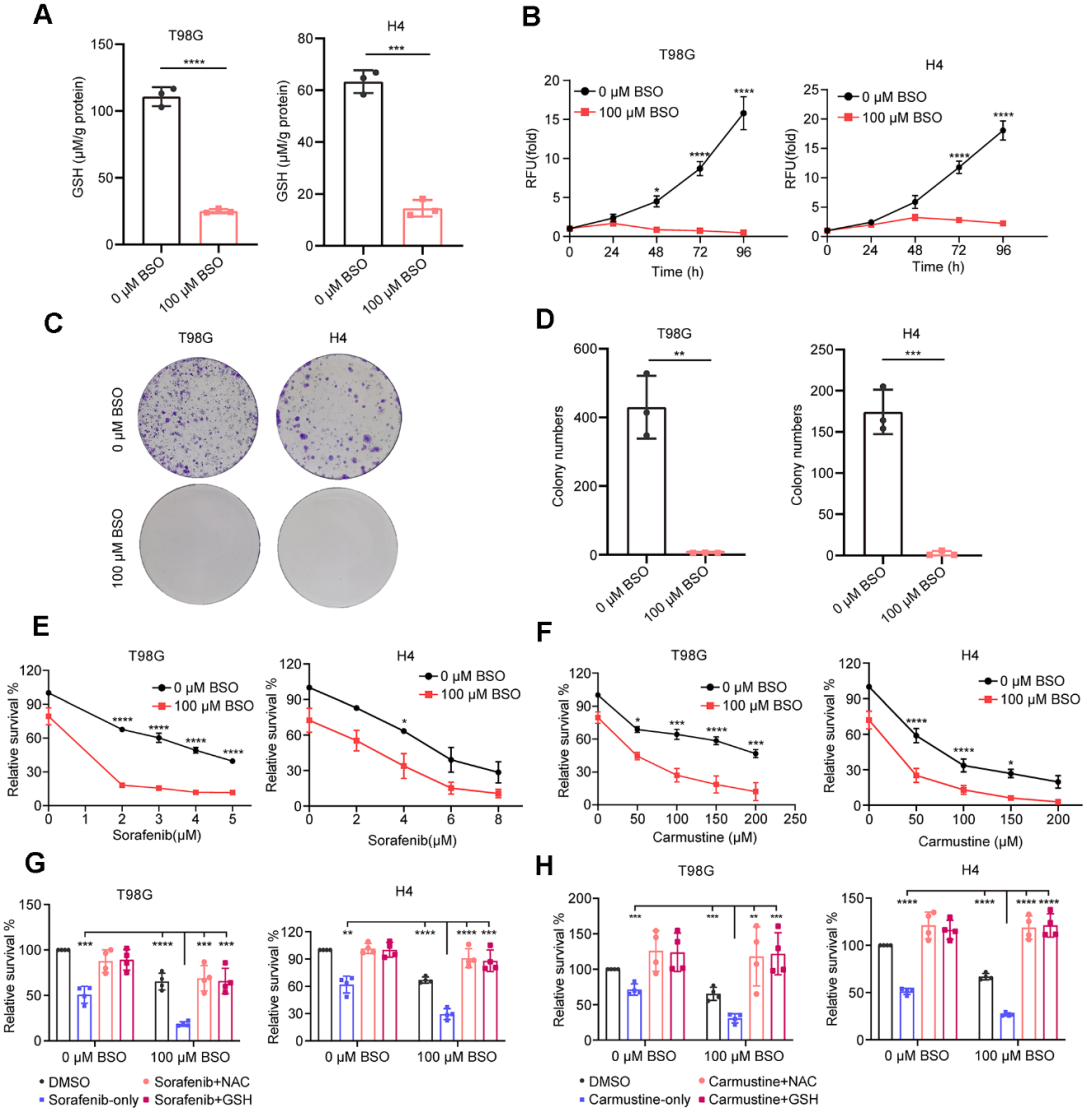
**Effect of decreased GSH levels on glioma cell proliferation and drug resistance.** (**A**) Measurement of glutathione content in T98G and H4 cell lines following preincubation with 100 μM L-buthionine-S,R-sulfoximine (BSO) for 24 h. (**B**) Effect of BSO treatment on the proliferation of T98G and H4 cell lines. (**C**) Colony formation results in response to BSO treatment. (**D**) Histograms showing the number of colonies under different experimental conditions. (**E**, **F**) Measurement of cell viability in T98G and H4 cells treated with varying concentrations of sorafenib or carmustine, either in combination with PBS or BSO (100 μM), for 24 h. (**G**, **H**) Cell viability assessment in T98G and H4 cells treated with BSO (100 μM) and sorafenib (4 μM) or carmustine (100 μM for T98G, 50 μM for H4), along with NAC (5 mM) or GSH (5 mM), for 24 hours. All data points are presented as mean ± SD from three or four independent experiments.

We selected one neuroglioma cell line, H4, and one glioblastoma cell line, T98G, since our GS also shows predictive power in the prognosis of glioblastoma patients (Supplementary Figure 4J). The selection of these cell lines aligns with the clinical observation that over 70% of LGG advance to higher-grade glioma or become aggressive within a decade [49]. Depleting glutathione using the glutathione synthesis inhibitor BSO [50] led to decreased GSH levels in both glioma cell lines (Figure 7A) and a significant reduction in cell proliferation and colony formation (Figure 7B–7D).

This treatment also enhanced glioma cells**’** drug sensitivity to sorafenib and carmustine (Figure 7E, 7F), consistent with our analysis showing that a low GS score is associated with higher sensitivity (lower sensitivity score) to these two drugs (Figure 5F). Additionally, the enhanced sensitivity can be completely reversed by the exogenous addition of antioxidants GSH and NAC (Figure 7G, 7H). These findings underscore the crucial role of glutathione metabolism in glioma proliferation and its significance in conferring resistance to chemotherapy treatments.

### NFE2L2 promotes glioma cell proliferation and migration

**Figure 8:**
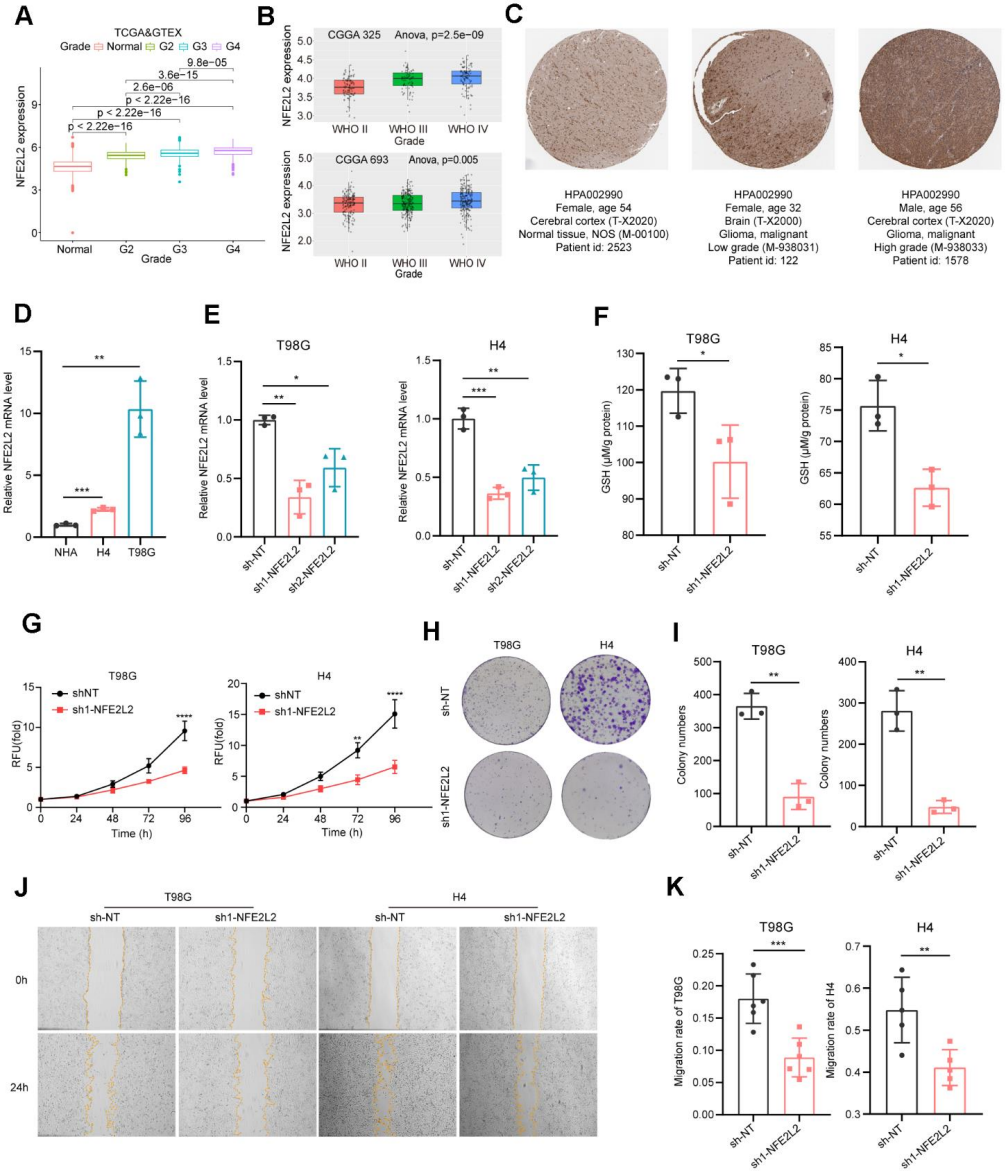
**Expression analysis and biological function assessment of NFE2L2.** (**A**) NFE2L2 expression in normal tissues and gliomas was examined using TCGA and GTEx data. (**B**) Quantification of NFE2L2 expression levels from CGGA datasets. (**C**) Typical IHC images of NFE2L2 expression in the HPA database. (**D**) qRT-PCR analysis of NFE2L2 mRNA expression in human astrocytes (NHA) and glioma cell lines (H4, T98G). (**E**) Validation of NFE2L2-specific shRNA knockdown efficiency in T98G and H4 cell lines by qRT-PCR. (**F**) Assessment of glutathione levels in T98G and H4 cells after NFE2L2 knockdown. (**G**) Impact of NFE2L2 knockdown on the proliferation of T98G and H4 cells. (**H**) Colony formation in response to NFE2L2 knockdown in T98G and H4 cell lines. (**I**) Histograms showing the number of colonies. (**J**) Scratch experiments revealing the effect of NFE2L2 knockdown on the migratory capacity of T98G and H4 cell lines. (**K**) Histograms showing the migration rate (N=5 or N=6). All data points were presented as mean ± SD, from three to six independent experiments.

Among the twelve signature genes in the risk score, NFE2L2 has the most prominent role (Figure 3B). The human gene NFE2L2 encodes nuclear factor erythroid 2-related factor 2 (NRF2), a master transcription factor against oxidative damage [51, 52]. NFE2L2 expression was significantly upregulated with increasing glioma grade (Figure 8A, 8B). This observation was further validated by pathology immunohistochemical staining, which revealed higher NFE2L2 protein levels in the patient samples with higher grade gliomas (Figure 8C).

Our experimental data demonstrated that glioma cell lines H4 and T98G exhibited higher expression levels of NFE2L2 compared to normal human astrocytes (NHAs) (Figure 8D). Additionally, NFE2L2 expression positively correlated with malignancy, with the glioblastoma cell line T98G showing significantly higher NFE2L2 levels than the neuroglioma cell line H4 (Figure 8D). Knockdown of NFE2L2 using shRNA (Figure 8E) resulted in reduced GSH levels in T98G and H4 cells (Figure 8F), along with significantly inhibited cell proliferation and clone formation capability in both cell lines (Figure 8G–8I). Moreover, the wound healing assay demonstrated a decrease in cell migration ability following NFE2L2 knockdown (Figure 8J, 8K). These findings further solidify the role of NFE2L2 as a key signature gene in our GS prediction system for LGG prognosis.

## DISCUSSION

Our investigation elucidates the role of glutathione metabolism, as reflected by the expression levels of a cluster of GMRGs, in predicting prognosis and treatment efficacy in LGG. Using GMRGs, both unsupervised clustering and a 12-core GMRGs-based risk model successfully categorized patients, revealing significant differences in cancer progression and treatment outcomes. Importantly, the risk model emerged as an independent prognostic factor, surpassing established models, and exhibited strong correlations with prognosis, proto-oncogene signaling, clinicopathology, and immune infiltration. Both methods demonstrated interrelatedness with respect to glutathione metabolism and were correlated with each other.

In experiments, we validated that depleting glutathione (GSH), either through the application of the chemical inhibitor BSO or by knocking down its regulatory gene NFE2L2, negatively impacted glioma cell proliferation, colony formation, and migration. These findings strongly suggest that glutathione metabolism likely has a direct effect on glioma cancer cells rather than acting through the manipulation of TIME.

However, our analysis reveals that the immune environment can also play an important supportive role, as indicated by our risk score model**’**s association with differential immune landscape and immunotherapy prognosis. Despite the presence of tight junctions in the blood-brain barrier, functional lymph nodes and various immune cell types exist within the CNS [53–55]. In brain tumors, the majority of immune cells are macrophages [56], predominantly of the immunosuppressive M2 subtype, which contribute to an immunosuppressive role by upregulating PD-L1 expression [57–59]. Notably, the high-risk group in our study exhibited significantly higher levels of macrophage infiltration, potentially fostering an immunosuppressive environment. Consistent with previous findings, our analysis suggests that heightened macrophage infiltration is detrimental to the patient**’**s prognosis.

Both reducing and increasing ROS levels have been explored as potential therapeutic strategies, and the choice depends on the specific cancer type and stage. Our study, incorporating both database analyses and experimental findings, suggests that inducing a more oxidized environment by inhibiting glutathione (GSH) synthesis can impede glioma growth. This effect may be due to distinct metabolic signatures associated with this cancer type, elucidating the effectiveness of our GMRG-based risk score in predicting prognosis for low-grade glioma (LGG) patients (Supplementary Figures 1, 4J). Indeed, lower grade gliomas and astrocytomas are reported to exhibit higher GSH levels than their higher-grade counterparts [60, 61]. Whether this risk score model could translate into glutathione antioxidant treatment is not clear. To establish consistent clinical outcomes with antioxidant treatments in brain cancers, comprehensive mechanistic studies are essential, considering the tumor state, stages, and specific brain region locations.

In summary, our study extensively investigated the link between GMRGs and LGG prognosis. By establishing a scoring system to quantify the GMRGs-based risk, the score could serve as a valuable biomarker for predicting prognosis and response to immunotherapy and chemotherapy, which could contribute to the progress of precision therapies and clinical management of LGG patients.

## MATERIALS AND METHODS

### Data acquisition and pre-processing

The Cancer Genome Atlas (TCGA)-LGG (N=514) gene expression data (FPKM), somatic mutations, and clinical information were obtained from the GDC database (https://portal.gdc.cancer.gov/). The data used to analyze differential expression of glutathione metabolism genes between LGG and normal brain tissues were downloaded from UCSC XENA, where batch effects have been removed [62]. The expression data for 33 cancer types and gene copy number variants (CNVs) for TCGA-LGG were also downloaded from UCSC XENA (https://xenabrowser.net/datapages/). The validation cohort (N=420) (DataSet ID: mRNAseq_693) was acquired from the Chinese Glioma Genome Atlas (CGGA) database. The expression level and perturbation effect of the 12 model genes in CNS/brain cell lines were downloaded from the DepMap database (https://depmap.org/portal/). The IMvigor210 dataset (N=348) was downloaded using the R package “IMvigor210CoreBiologies”. TCGA-LGG mutation annotation format (MAF) files were analyzed using the R package “maftools”.

### GMRGs consensus clustering

Consensus clustering of LGG patients based on GMRG expression was performed using the R package “ConsensusClusterPlus” [63, 64]. Euclidean distance clustering (K=2) was performed on 80% of the samples resampled 50 times. Kaplan-Meier (K-M) survival analysis was used to compare the prognosis differences between the two clusters [64, 65].

### Differential gene screening and enrichment analysis

Genes with a log fold change greater than 1 and an FDR less than 0.05 between the two clusters were considered differentially expressed. These genes were then used for GO and KEGG enrichment analysis using the “Clusterfiler” R package [66, 67].

### Development and validation of GMRGs-based risk score

The TCGA-LGG and CGGA-LGG datasets were standardized to remove batch effects, followed by univariate Cox regression analysis of shared GMRGs to identify prognostic genes. These genes were subjected to three machine learning algorithms (Lasso, SuperPC, and plsRcox) to select for the optimal combination for constructing the risk signature. Eventually, these genes were subjected to LASSO regression for further analysis. Risk scores were calculated as the sum of (gene coefficient * gene expression). Based on the median risk score of the TCGA-LGG dataset, the TCGA-LGG and CGGA-LGG datasets were categorized into high-risk and low-risk groups. Kaplan-Meier analysis was used to compare prognostic differences between the groups. The relationship between the risk signature and clinicopathological characteristics was analyzed using the “ggpubr” package. The independence of the risk signature as a prognostic factor was assessed using the “survival” and “survminer” packages. The predictive ability of the risk signature was evaluated and compared to other systems using the “survival”, “survminer”, and “survcomp” packages. The receiver operating characteristic (ROC) curves of the GS model and some clinical factors were plotted with the “survival”, “survminer”, and “timeROC” packages.

### Functional enrichment analysis

Gene Set Variation Analysis (GSVA) was performed on gene sets “c2.cp.kegg.v2023.1.Hs.symbols.gmt” and “h.all.v2023.1.Hs.symbols.gmt” using the R package “GSVA”, and Gene Set Enrichment Analysis (GSEA) was performed on gene set “c6.all.v2023.1.Hs.symbols.gmt” using the R package “clusterProfiler”. Glutathione metabolism scores were calculated using Single Sample Gene Set Enrichment Analysis (ssGSEA) on the gene set “GOBP_GLUTATHIONE_METABOLIC_PROCESS”, and differences between groups were evaluated using the “ggpubr” package. The gene sets used above were obtained from the Molecular Signature Database (MsigDB, http://software.broadinstitute.org/gsea/msigdb/). IHC images for NFE2L2 were obtained from the HPA database (https://www.proteinatlas.org/).

### Immune landscape identification and immunotherapy assessment

The ESTIMATE algorithm was used to assess StromalScore, ImmuneScore, and ESTIMATEScore in LGG tumor microenvironment. Immune cell infiltration analysis (TIMER, CIBERSORT, quanTIseq, xCell, MCP-counter, EPIC) [68–73] were obtained from TIMER2 online tool (http://timer.cistrome.org/) and used in Spearman correlation analysis and K-M analysis. Tumor Immune Dysfunction and Exclusion (TIDE) scores of the TCGA-LGG samples were generated using the TIDE online tool (http://tide.dfci.harvard.edu/). Prognostic analysis of glutathione metabolism genes across 33 cancer types were performed using the Gene Set Cancer Analysis (GSCA) online platform (http://bioinfo.life.hust.edu.cn/GSCA/#/). Cancer-Immunity Cycle data were sourced from the TIP website (http://biocc.hrbmu.edu.cn/TIP/help.jsp), and a list of immune inhibitors was obtained from TISIDB (http://147.8.185.80/TISIDB/). TIDE scores, Cancer-Immunity Cycle activity scores, and immune inhibitor genes expression levels were compared using the “ggpubr” package.

### Analysis of drug sensitivity

The drug sensitivity score for chemotherapeutic agents in LGG patients was predicted using the R package “oncopredict”, with expression profile data and corresponding drug response information sourced from the GDSC2 database. Differences in drug sensitivity between the high-risk and low-risk groups were examined using the Wilcox test.

### Single-cell sequencing analysis and construction of nomograms

Gene expression in the Glioma_GSE89567, Glioma_GSE70630, and Glioma_GSE131928_10X single-cell datasets were analyzed using the TISCH2 website (http://tisch.comp-genomics.org/home/) [74]. A nomogram was generated by integrating gene signatures and clinical characteristics with the R package “RMS”. Each patient**’**s total score was calculated as the sum of variable scores for each parameter. The calibration curves were used to assess the prognostic value of the predicted 1-, 3-, and 5-year OS rates in comparison to actual observations.

### Cell culture

The NHA, T98G, H4 and HEK-293T cell lines were obtained from the American Type Culture Collection (ATCC) and cultured in DEME medium (Cytiva) supplemented with 1% penicillin/streptomycin and 10% FBS (Gibco). These cells were maintained at 37° C in a humidified atmosphere containing 5% CO2.

### GSH concentration measurement

The cells were trypsin-digested, washed thrice with PBS, and subsequently resuspended in PBS before undergoing sonication. Protein concentrations of the samples were measured using the BCA protein assay kit (Beyotime Biotechnology), while GSH concentrations were measured using the reduced glutathione assay kit (Nanjing Jiancheng Bioengineering Institute). The GSH concentration of the samples was then normalized to the protein concentration.

### Cellular assays

In cell viability assays, H4 and T98G cells were seeded at 2000 cells per well in 96-well plates and treated with varying concentrations of L-buthionine-S,R-sulfoximine (BSO), sorafenib, or carmustine. After 24 hours, cell viability was measured using 10 μg/ml Resazurin. For colony formation, H4 and T98G cells were plated at 2000 cells per well in six-well plates and the medium changed every three days over 11 days. Subsequently, the cells were fixed, stained with crystal violet, and analyzed using ImageJ. In the wound healing assay, cells were seeded at 3x10^5 cells per well in a six-well plate with a straight line marked at the bottom. The following day, a sterile 200 μl tip created a perpendicular scratch, and after three PBS washes, serum-free medium was added. Photographs at 0 and 24 hours recorded the scratch positions, and cell migration was analyzed with ImageJ.

### Lentiviral packaging and gene knockdown

The shRNA plasmid, along with pCMV-VSV-G, psPAX2 plasmids, were transfected into HEK-293T cells with PEI 40K (Shanghai Maokang Biotechnology). After 15h, the medium was replaced, and the virus was collected at 48 and 72 hours. The harvested virus was centrifuged at 1000 rpm for 3 minutes and filtered through a 0.48 μm filter. To knock down the gene in glioma cells, 1 ml of virus was added to T98G and H4 cells, with the addition of Polybrene (Beyotime Biotechnology) at a final concentration of 8 μg/ml. After 24 hours of virus infection, the medium was changed, and the cells were selected with 3 μg/ml puromycin. The knockdown efficiency was subsequently assessed via qRT-PCR. The targeted sequences for knockdown were as follows:

sh1-NFE2L2: CCGGCATTTCACTAAACACAA

sh2-NFE2L2: GCTCCTACTGTGATGTGAAAT

### RNA extraction and qPCR

Cells were lysed using RNAiso Plus (Takara), and RNA was extracted using standard phenol-chloroform extraction method. Subsequently, 1 μg of RNA was reverse transcribed into cDNA using HiScript III RT SuperMix for qPCR (Vazyme) and then subjected to qPCR with Taq Pro Universal SYBR qPCR Master Mix Q712 (Vazyme). ACTB served as an internal reference for normalization. Gene expression levels were quantified using the 2-ΔΔCt method. The qPCR primers utilized were:

NFE2L2-FW: TCAGCGACGGAAAGAGTATGA

NFE2L2 -RV: CCACTGGTTTCTGACTGGATGT

ACTB-FW: CATGTACGTTGCTATCCAGGC

ACTB-RV: CTCCTTAATGTCACGCACGAT

### Data visualization and statistical analysis

All analyses and graphs were generated using R software (R version 4.2.3) and GraphPad Prism 8.0.2. The “limma” R package was used to identify differential genes. Kaplan-Meier (K-M) survival analysis was conducted using log-rank tests. Correlation analysis employed the Spearman or Pearson methods. Differences between the two groups were assessed using the Wilcoxon test and unpaired t-tests. Multiple comparisons in cell viability as a function of time or increasing drug concentrations were calculated using one-way ANOVA with post hoc Sidak test. P<0.05 was considered significant (*), p<0.01 (**), p<0.001 (***), and p<0.0001 (****).

### Data availability

Public data used in this work can be acquired from the TCGA Research Network portal (https://portal.gdc.cancer.gov/) and Chinese Glioma Genome Atlas (CGGA, http://www.cgga.org.cn/). All codes are available on GitHub: https://github.com/YuBestLab/YuBestLab.github.io/tree/master.

## Supplementary Material

Supplementary Figures

Supplementary Table 1

Supplementary Table 2

Supplementary Table 3

Supplementary Table 4
