# Supplementary material for: Glutathione metabolism-related gene signature predicts prognosis and treatment response in low-grade glioma
Source: Aging (Albany NY). 2024 May 30;16(11):9518–46. doi: 10.18632/aging.205881 (PMC11210255; doi:10.18632/aging.205881)
Supplement: Supplementary Figures [file aging-16-205881-s001.pdf]

SUPPLEMENTARY FIGURES

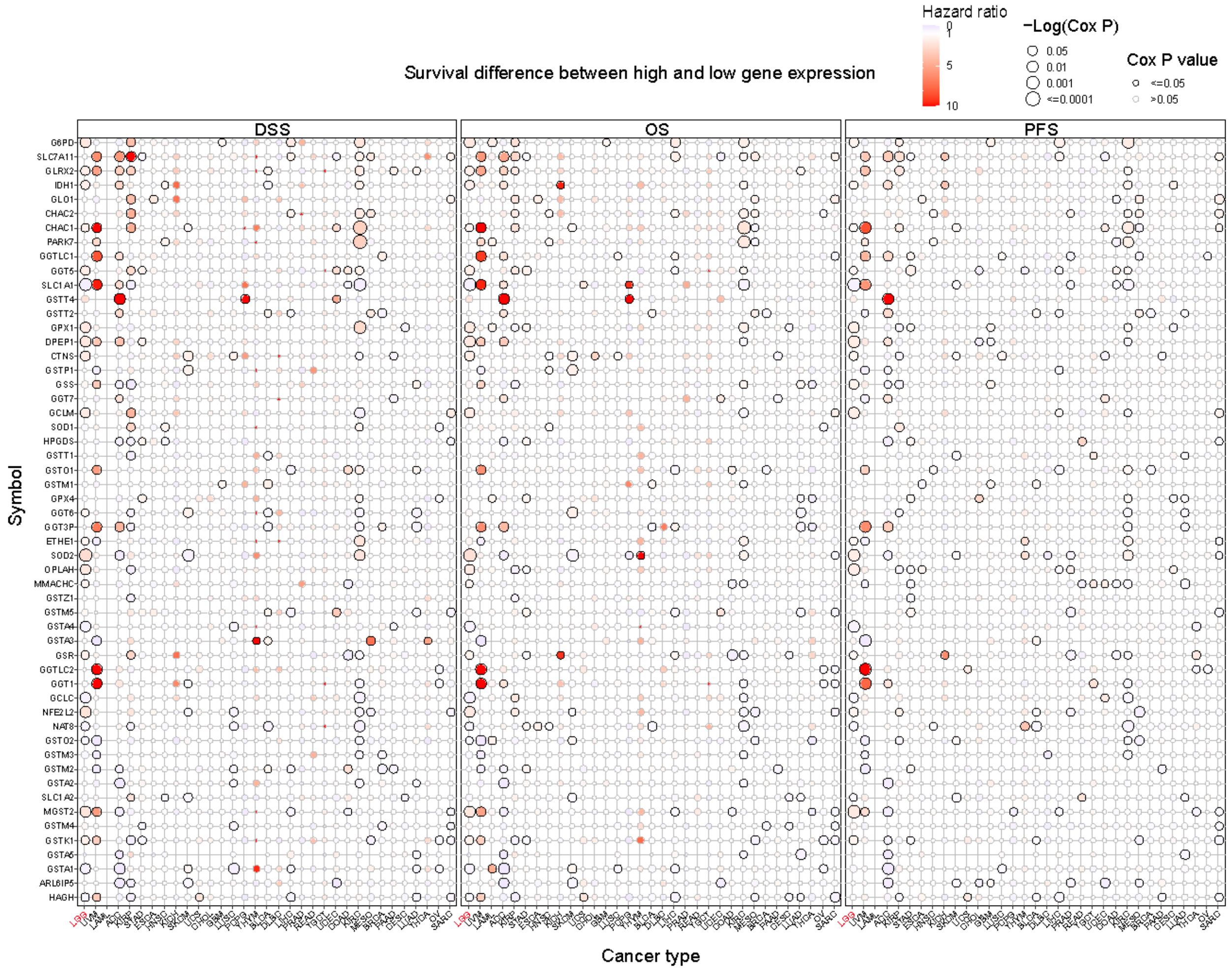

Supplementary Figure 1. Survival analysis of glutathione metabolism genes across 33 cancer types using the GSCA website.

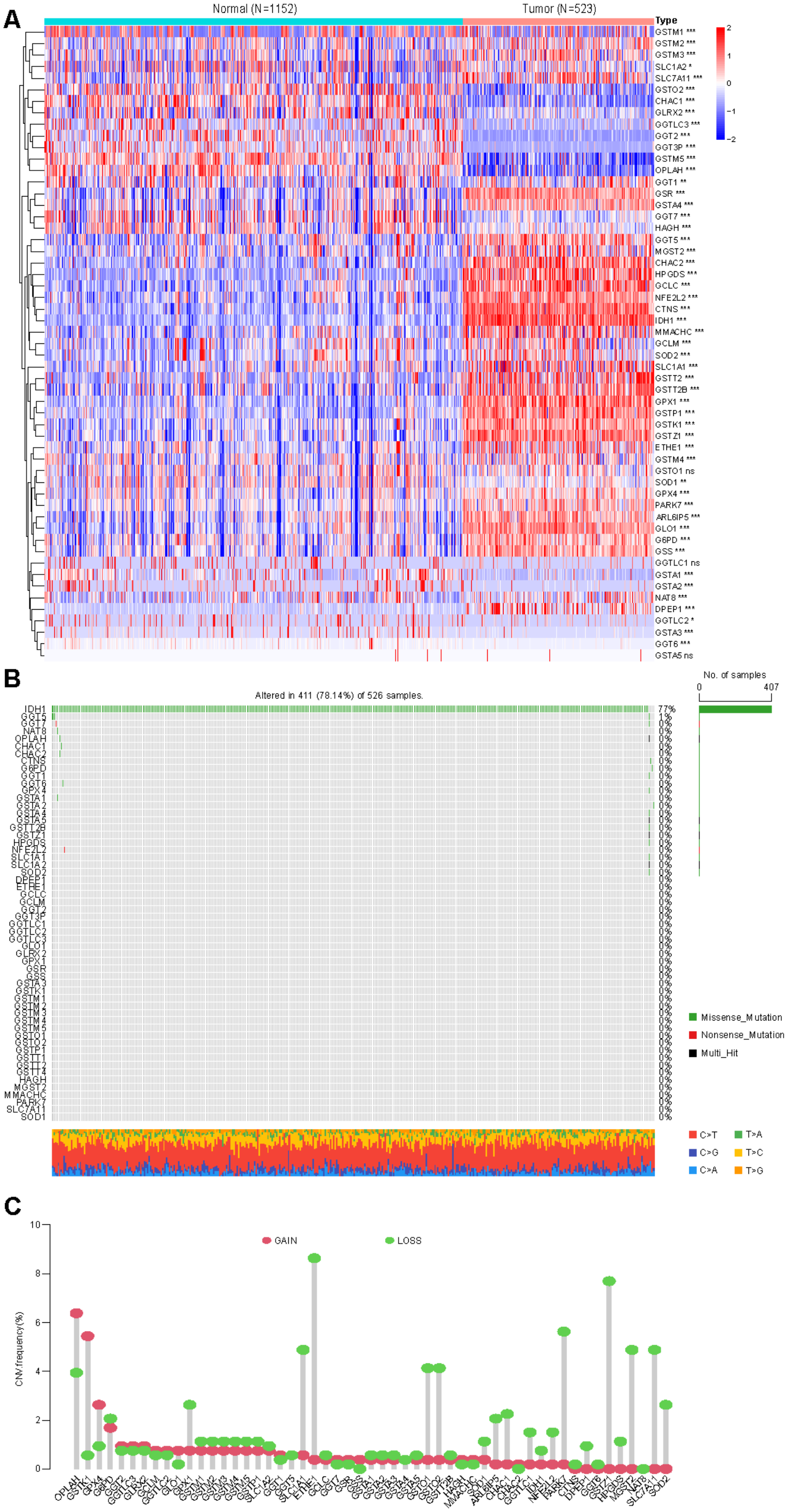

Supplementary Figure 2. Expression levels and genetic variation landscape of GMRGs. (**A**) Comparative expression levels of 56 GMRGs between LGG cancer and normal brain tissues. (**B**) Mutation frequency of GMRGs in patients from the TCGA-LGG dataset. (**C**) Copy number variation (CNV) frequency of GMRGs in the TCGA-LGG dataset.

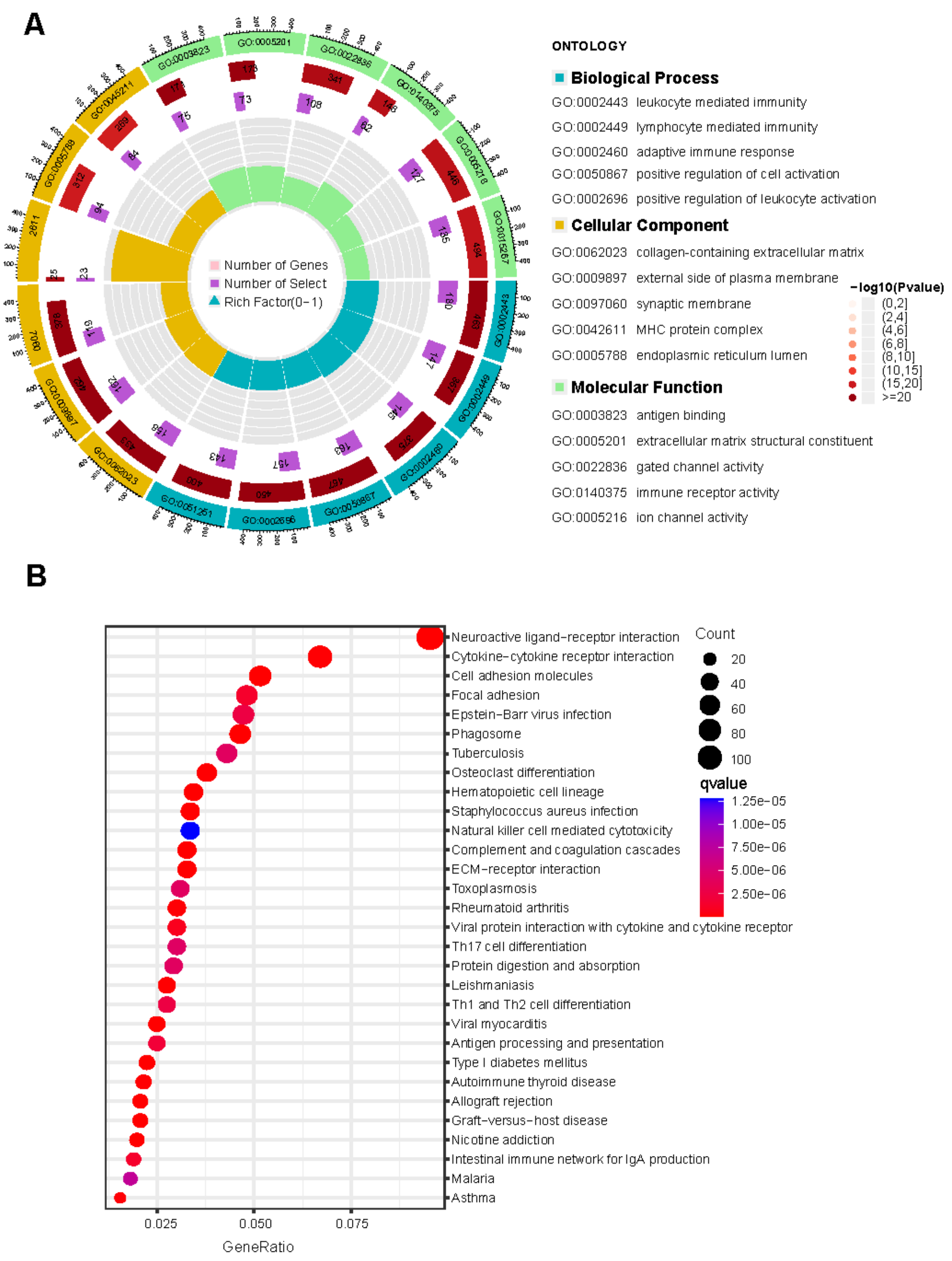

Supplementary Figure 3. Functional enrichment analysis of GMRGs-related clusters. (**A**) Gene Ontology (GO) enrichment analysis based on the Differentially Expressed Genes (DEGs). (**B**) Kyoto Encyclopedia of Genes and Genomes (KEGG) enrichment analysis based on the DEGs.

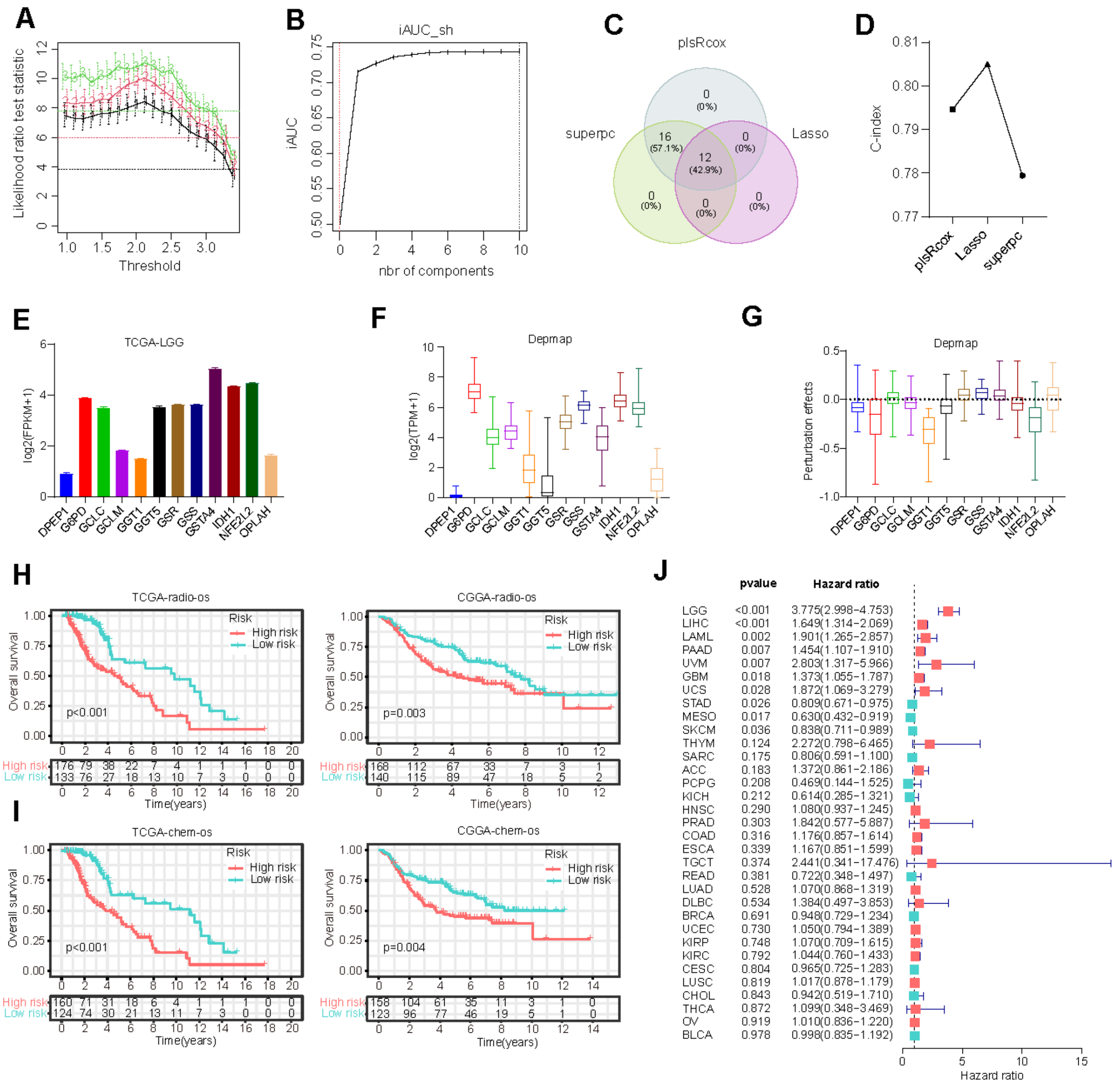

Supplementary Figure 4. Construction and confirmation of GMRGs-based risk score. (**A**) Cross-validation based on the SuperPC model. (**B**) Cross-validation based on the plsRcox model. (**C**) The Venn diagram displays the intersection of model genes from the three machine learning algorithms. (**D**) The C-index of the three machine learning algorithms. (**E**) Expression levels of model genes in TCGA-LGG dataset (**F**) Expression levels of model genes in the CNS/brain cell lines in the CCLE database. (**G**) Gene perturbation effects of model genes in the CNS/brain cell lines in the CCLE database. (**H**, **I**) OS curves for patients in high-risk and low-risk groups receiving radiation therapy or chemotherapy in TCGA-LGG or CGGA-LGG datasets. (**J**) Univariate Cox regression analysis based on the risk score across 33 kinds of cancer types.

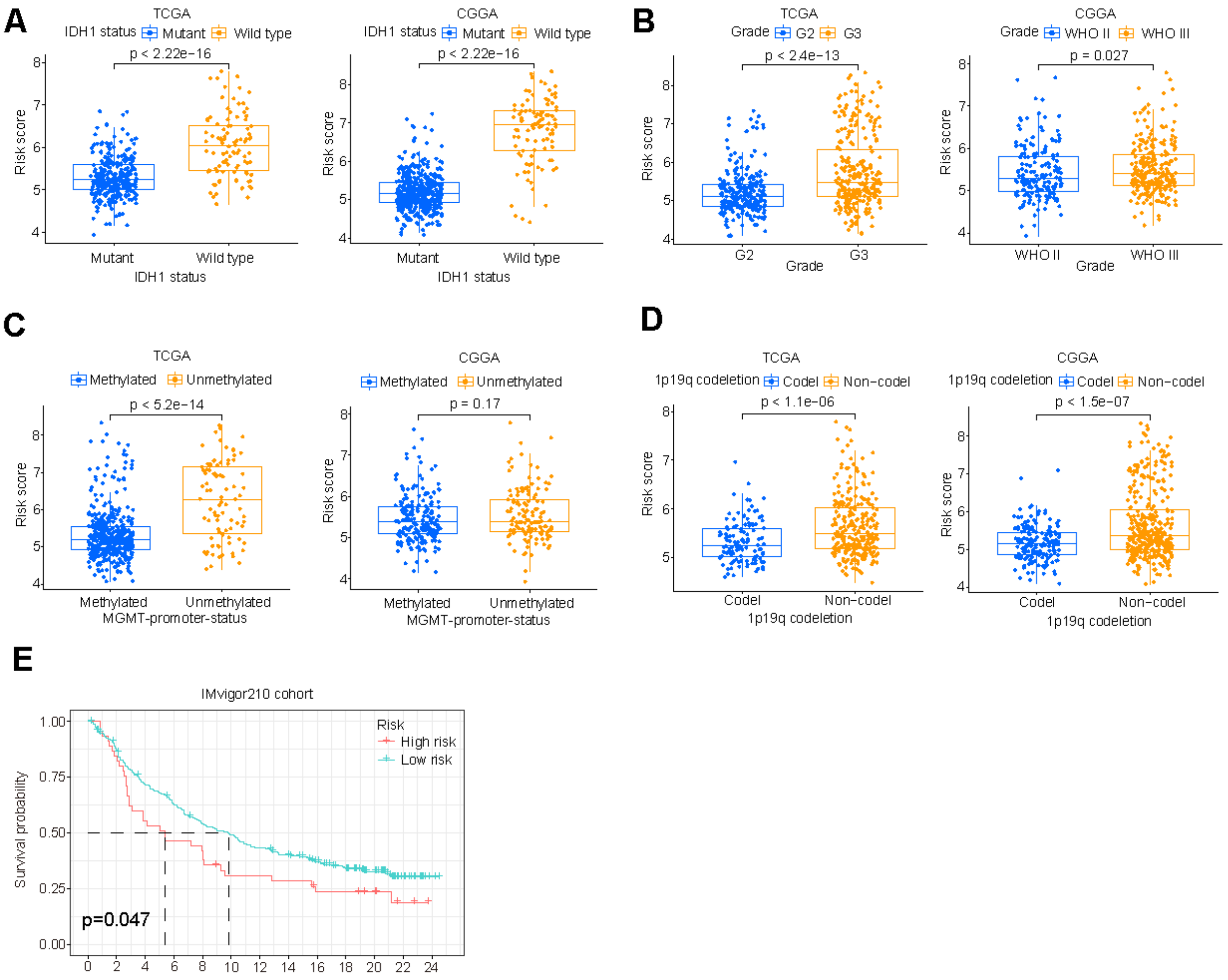

Supplementary Figure 5. Association of GS with clinical characteristics of LGG. (**A**–**D**) The risk scores are associated with clinical characteristics of IDH1 status (**A**), Grade (**B**), MGMT promoter status (**C**), and 1p19q co-deletion (**D**) in TCGA-LGG and CGGA-LGG datasets. (**E**) Survival analysis of the high-risk and low-risk groups in the immunotherapy IMvigor210 cohort.

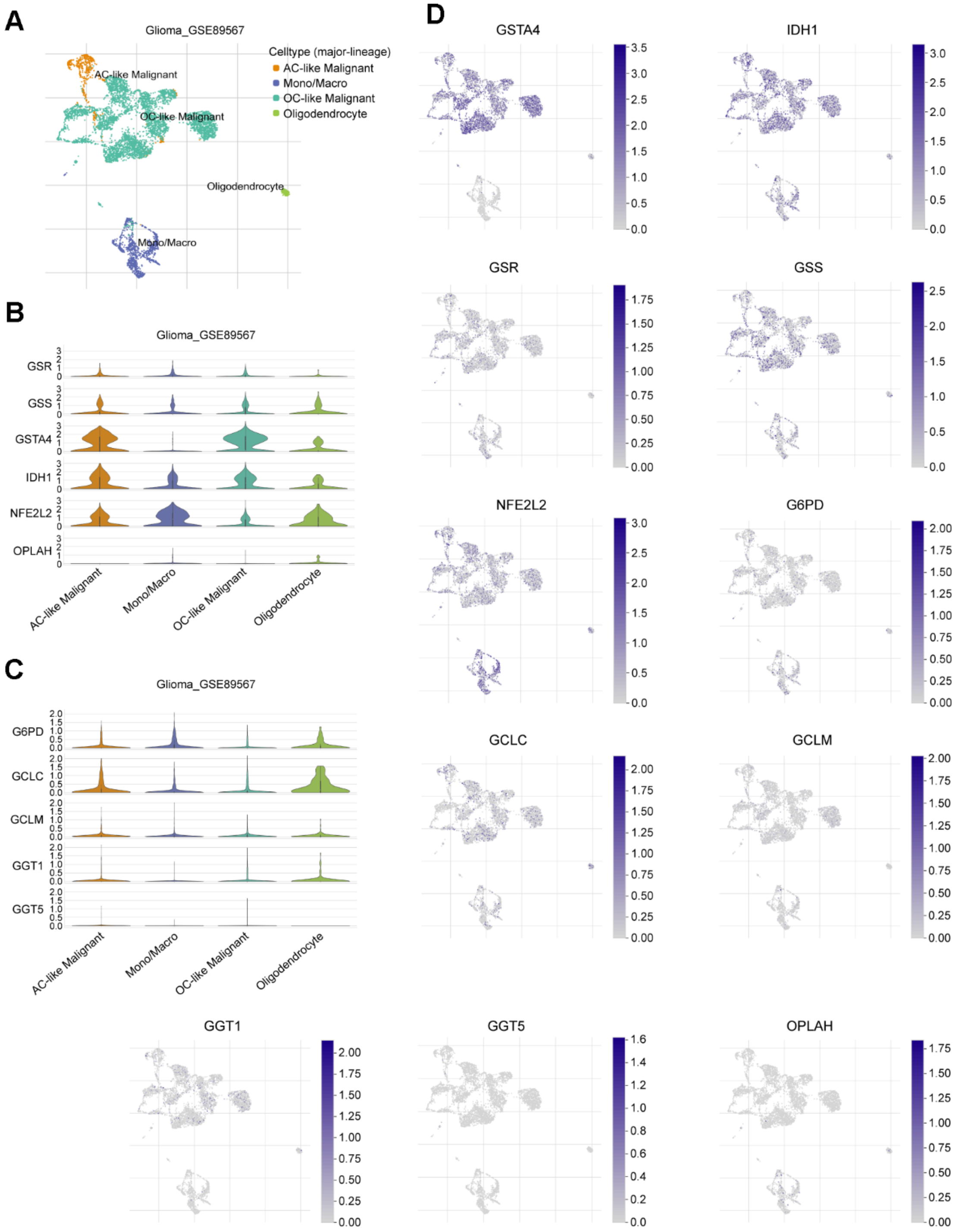

Supplementary Figure 6. Model gene expression patterns at the single-cell level. (**A**) Identification of major cell subtypes in the LGG single-cell dataset Glioma GSE89567. (**B**, **C**) Expression patterns of model genes across different cell populations. (**D**) Expression patterns of model genes in different cell populations.

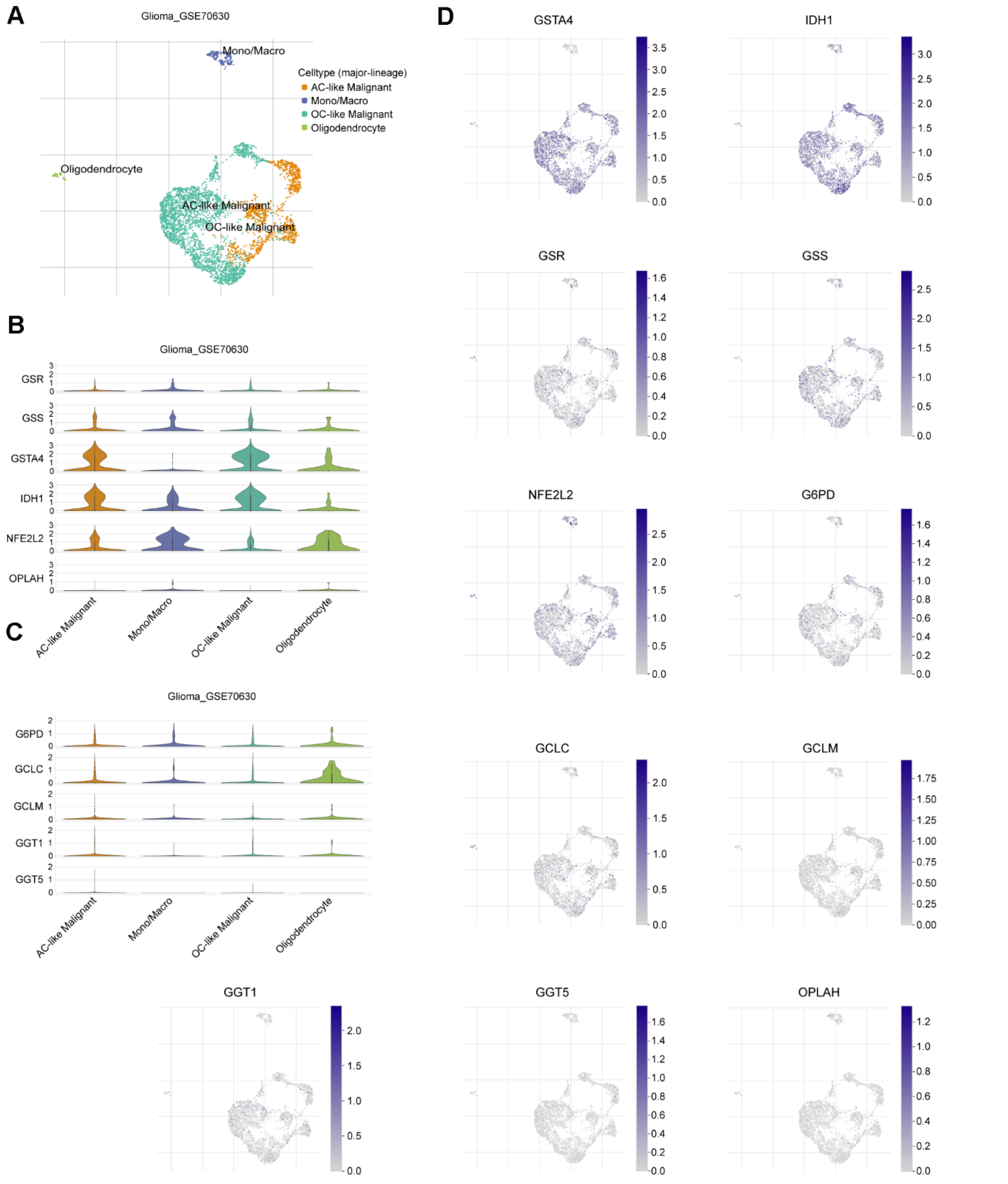

Supplementary Figure 7. Model gene expression patterns at the single-cell level. (**A**) Identification of major cell subtypes in the LGG single-cell dataset Glioma_GSE70630. (**B**, **C**) Expression patterns of model genes across different cell populations. (**D**) Expression patterns of model genes in different cell populations.

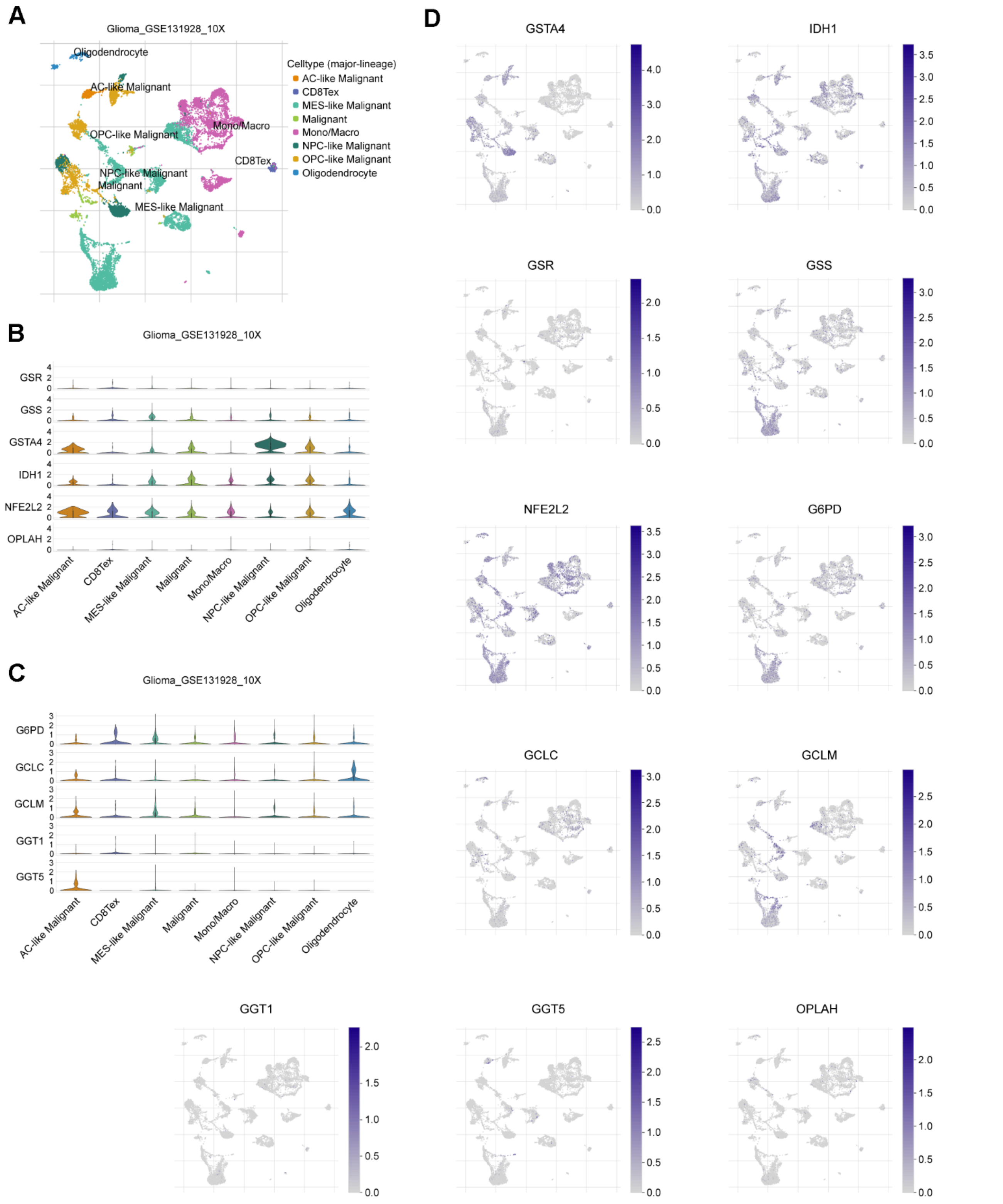

Supplementary Figure 8. Model gene expression patterns at the single-cell level. (**A**) Identification of major cell subtypes in the Glioma_GSE131928_10X single-cell dataset. (**B**, **C**) Expression patterns of model genes across different cell populations. (**D**) Expression patterns of model genes in different cell populations.
